# Erratum for Foxall et al., “*Inoviridae* prophage and bacterial host dynamics during diversification, succession, and Atlantic invasion of Pacific-native *Vibrio parahaemolyticus*”

**DOI:** 10.1128/mbio.01020-24

**Published:** 2024-04-22

**Authors:** Randi L. Foxall, Jillian Means, Ashley L. Marcinkiewicz, Christopher Schillaci, Kristin DeRosia-Banick, Feng Xu, Jeffrey A. Hall, Stephen H. Jones, Vaughn S. Cooper, Cheryl A. Whistler

## ERRATUM

Volume 15, issue 1, e02851-23, 2024, https://journals.asm.org/doi/10.1128/mbio.02851-23. Page 1: Ashley L. Marcinkiewicz’s first name should appear as given in this Erratum.

