# Supplementary material for: Inoviridae prophage and bacterial host dynamics during diversification, succession, and Atlantic invasion of Pacific-native Vibrio parahaemolyticus
Source: mBio. 2023 Dec 19;15(1):e02851-23. doi: 10.1128/mbio.02851-23 (PMC10790759; doi:10.1128/mbio.02851-23)
Supplement: Supplemental figures — Figures S1 to S6. [file mbio.02851-23-s0001.pdf]

Figure S1

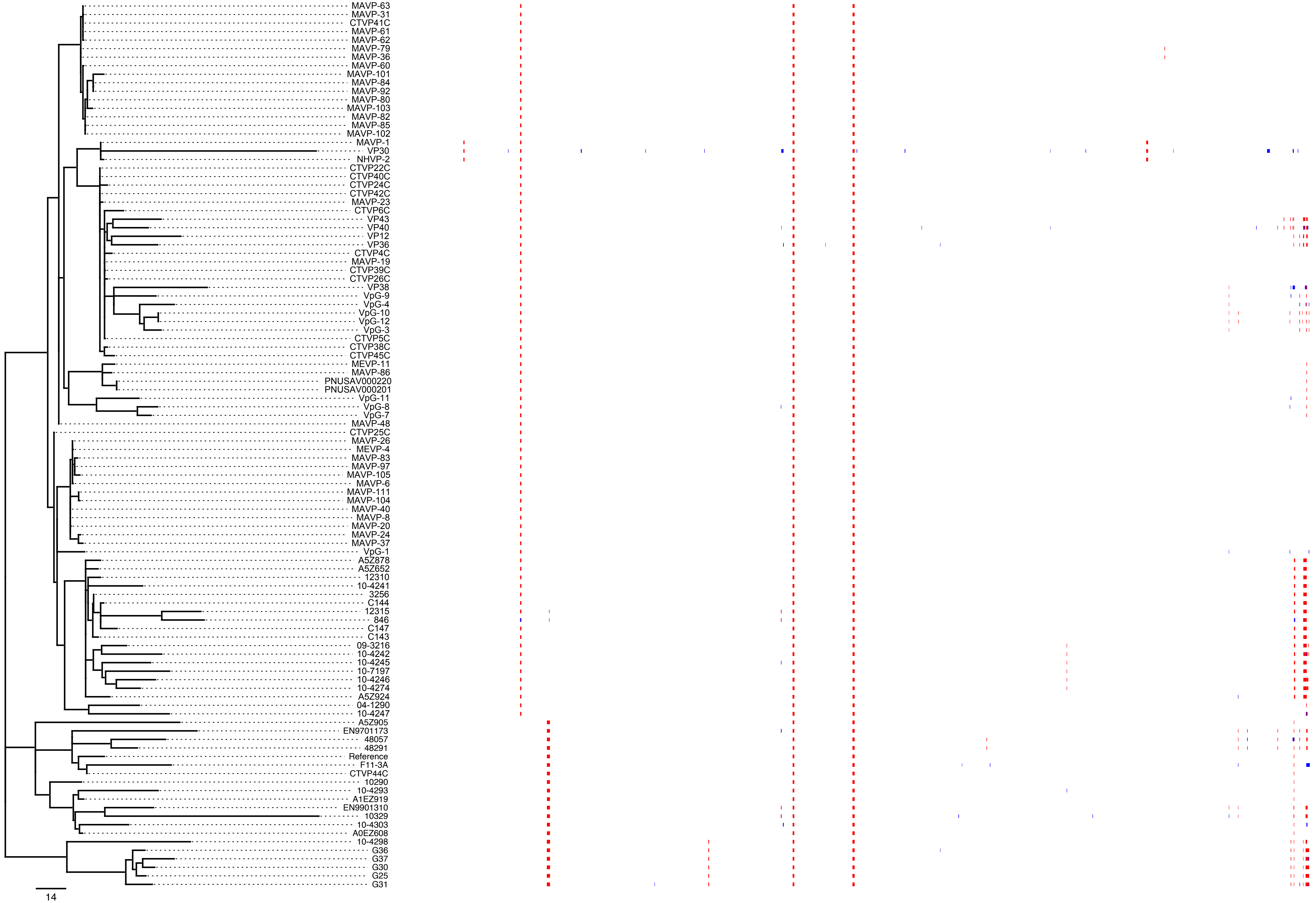

Supplemental Figure S1. Phylogeny of ST36 excluding regions of recombination as visualized by Gubbins. A maximum-likelihood (ML) phylogeny was built with 1,127 aligned SNPs identified in non-recombining regions (non-colored regions) and excluding regions of recombination exhibiting a higher SNP density (colored blocks) as identified by Gubbins (102) . Red blocks indicate recombination within a clade of related isolates, whereas blue blocks indicate recombination with isolates that were absent from the analysis. Analysis inadvertently excluded a few isolates included in whole genome phylogenies.

Figure S2

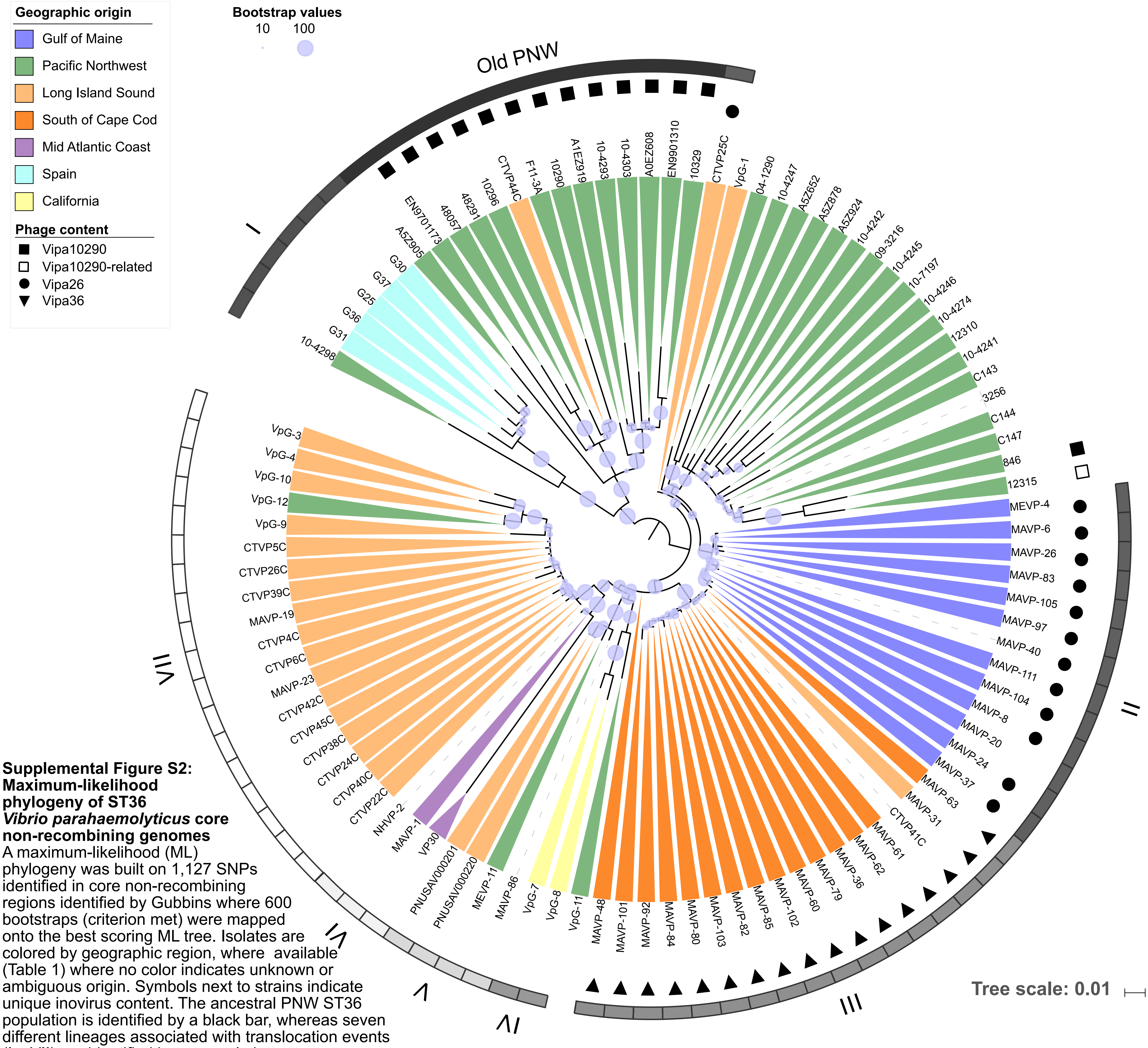

Supplemental Figure S2: Maximum-likelihood phylogeny of ST36 *Vibrio parahaemolyticus* core non-recombining genomes
A maximum-likelihood (ML) phylogeny was built on 1,127 SNPs identified in core non-recombining regions identified by Gubbins where 600 bootstraps (criterion met) were mapped onto the best scoring ML tree. Isolates are colored by geographic region, where available (Table 1) where no color indicates unknown or ambiguous origin. Symbols next to strains indicate unique inovirus content. The ancestral PNW ST36 population is identified by a black bar, whereas seven different lineages associated with translocation events (I – VII) are identified by greyscale bars.

Figure S3

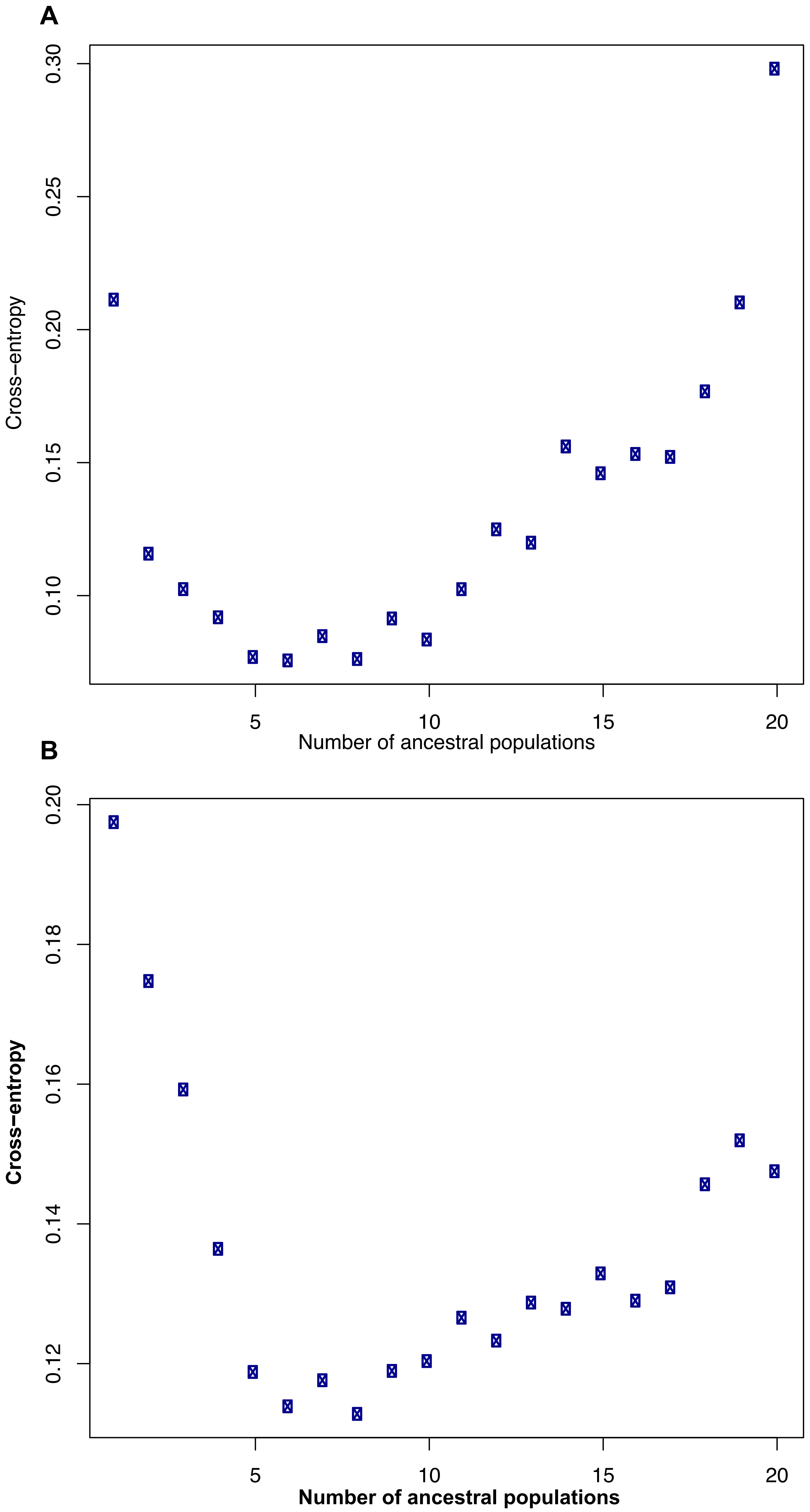

Supplemental Figure S3. Cross-entropy derived from the coancestry distribution for a sequentially increasing number of ancestors that contributed nucleotide variation in the ST36 population. Cross-entropy was determined by LEA (49) by empirical evaluation of the likelihood that between 1-20 ancestors explains the distribution of core, non-recombining (A) or whole (B) genome variation among the global population of ST36. The lowest entropy was observed at 6 and 8 (Fig. 2) ancestral populations for ore, non-recombining data, and 8 ancestral populations (see Fig. S4) for whole genome data.

Figure S4

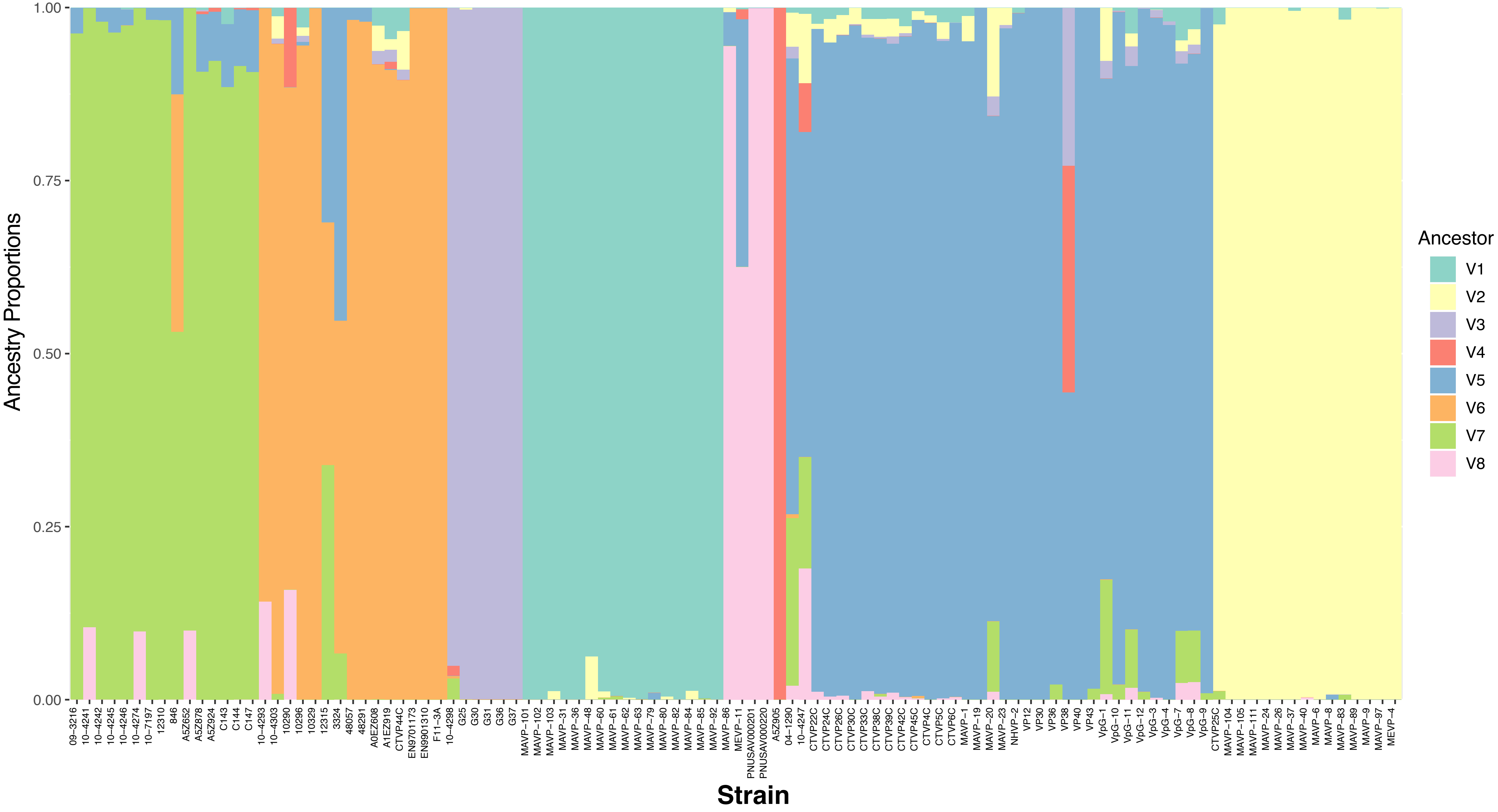

Supplemental Figure S4. Population structure of ST36 strains using whole genome variation as explained by eight unique ancestral populations. Coancestry estimates were inferred from matrices of 3545 SNPs generated from the whole genomes of ST36 strains by LEA (49). Colored bars represent proportions of genetic variation derived from eight ancestral populations which generated the lowest level of cross-entropy (see Fig. S3B).

Figure S5

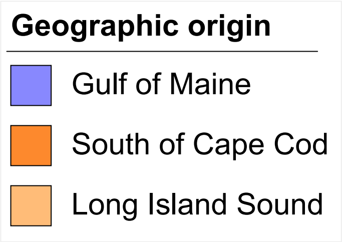

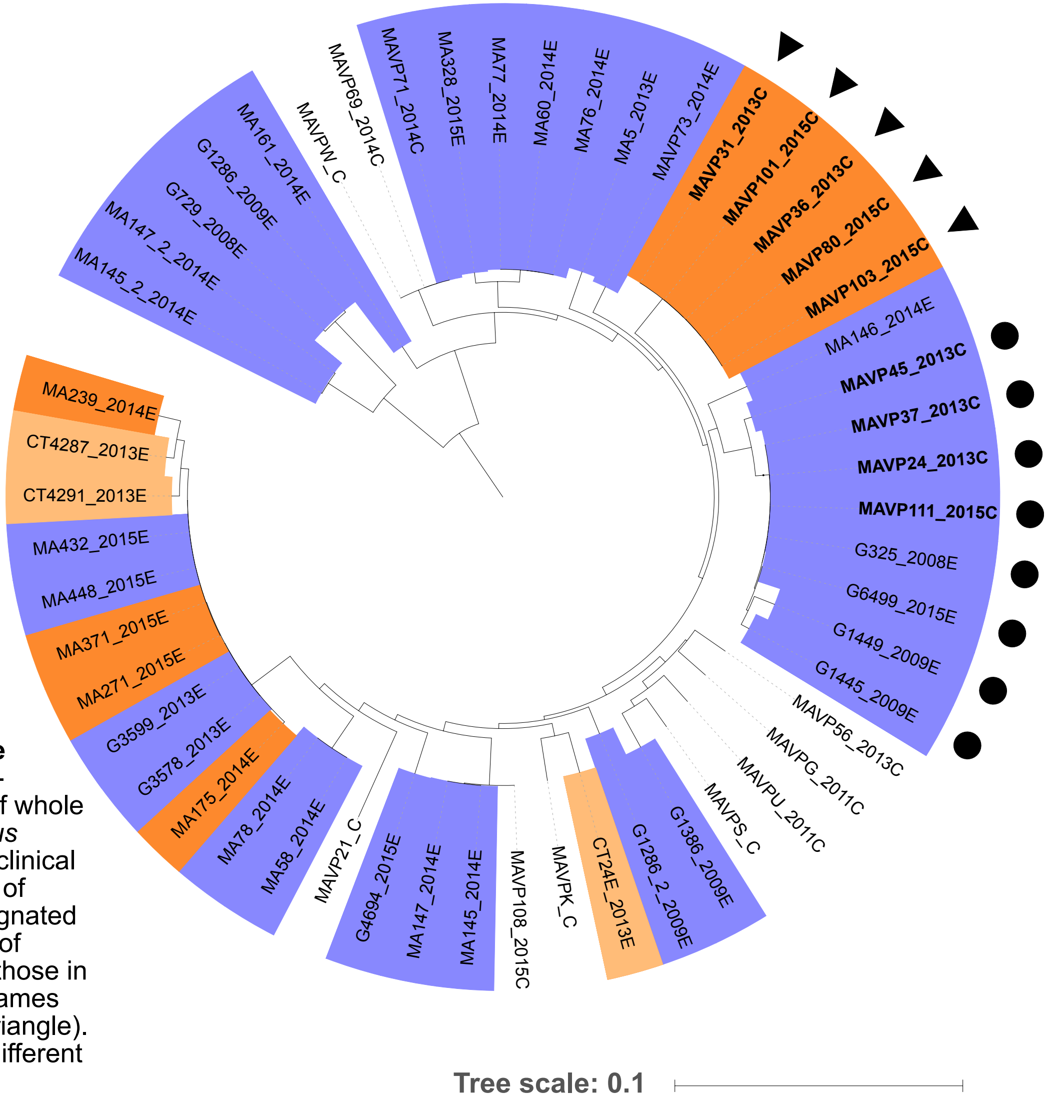

Supplemental Figure S5. Maximum Likelihood whole genome phylogenies of inoviruses. Genome-BLAST Distance Phylogeny (GBDP) of whole phage genomes of *V. parahaemolyticus* ST36 and other sequence types from clinical cases (C), and from the environments of GOM, SCC and LIS locations (E) designated following the year of isolation. Colors of environmental location correspond to those in Figure 1, and shapes next to isolate names highlight Vipa26 (circle) and Vipa36 (triangle). G1286, and MA145 each harbor two different unrelated phage.

Figure S6

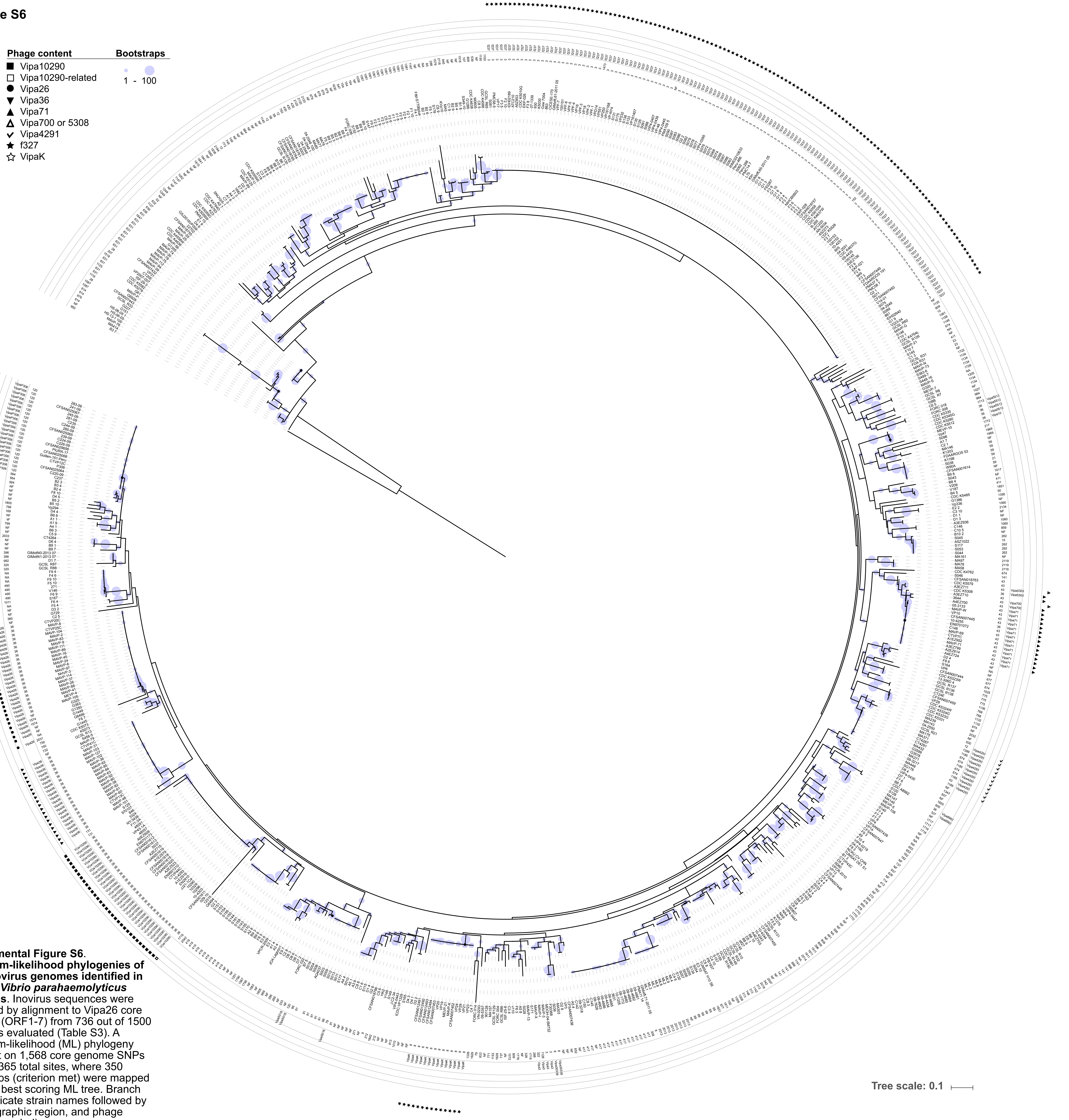

Supplemental Figure S6. **Maximum-likelihood phylogenies of core inovirus genomes identified in diverse *Vibrio parahaemolyticus* genomes**. Inovirus sequences were extracted by alignment to Vipa26 core genome (ORF1-7) from 736 out of 1500 genomes evaluated (Table S3). A maximum-likelihood (ML) phylogeny was built on 1,568 core genome SNPs out of 5,365 total sites, where 350 bootstraps (criterion met) were mapped onto the best scoring ML tree. Branch ends indicate strain names followed by ST, geographic region, and phage identity (symbol).
